# How to make an inclusive-fitness model

**DOI:** 10.1098/rspb.2023.1310

**Published:** 2023-10-04

**Authors:** Thomas W. Scott, Geoff Wild

**Affiliations:** ^1^ Department of Biology, University of Oxford, 11a Mansfield Road, Oxford OX1 3SZ, UK; ^2^ Department of Mathematics, Western University, 1151 Richmond Street, London, Ontario, Canada N6A 5B7

**Keywords:** Hamilton’s rule, informal Darwinism, kin selection, social evolution

## Abstract

Social behaviours are typically modelled using neighbour-modulated fitness, which focuses on individuals having their fitness altered by neighbours. However, these models are either interpreted using inclusive fitness, which focuses on individuals altering the fitness of neighbours, or not interpreted at all. This disconnect leads to interpretational mistakes and obscures the adaptive significance of behaviour. We bridge this gap by presenting a systematic methodology for constructing inclusive-fitness models. We find a behaviour’s ‘inclusive-fitness effect’ by summing primary and secondary deviations in reproductive value. Primary deviations are the immediate result of a social interaction; for example, the cost and benefit of an altruistic act. Secondary deviations are compensatory effects that arise because the total reproductive value of the population is fixed; for example, the increased competition that follows an altruistic act. Compared to neighbour-modulated fitness methodologies, our approach is often simpler and reveals the model’s inclusive-fitness narrative clearly. We implement our methodology first in a homogeneous population, with supplementary examples of help under synergy, help in a viscous population and Creel’s paradox. We then implement our methodology in a class-structured population, where the advantages of our approach are most evident, with supplementary examples of altruism between age classes, and sex-ratio evolution.

## 1. Introduction

Many biological traits are *social*, i.e. they have fitness consequences for other individuals in addition to themselves. To study social behaviours theoretically, we cannot get away with simply considering the fitness of the individual expressing the trait. Instead, we need to construct a *social evolution* model, which also considers the fitness of other affected individuals. Hamilton [1] showed that there are two alternative ways to construct a social evolution model. The first is the neighbour-modulated fitness approach (*aka* the direct fitness approach), which counts up fitness effects on a recipient of the behaviours of a number of actors (recipient-centric). The second is the inclusive-fitness approach, which counts up the fitness effects arising from a focal individual acting in an environment stripped of other social interactions (actor-centric). Under a standard set of mathematical assumptions, the two approaches are mathematically equivalent, in that they make the same predictions regarding whether a given social behaviour will be favoured [1,2].

Inclusive fitness, as a modelling paradigm, can be difficult to use, as it departs from the traditional population-genetics approach of counting up fitness effects on a focal individual in its experienced environment (John Maynard Smith called it ‘an absolute swine to calculate’; see [3]). This has additionally led to it being characterized as less mathematically rigorous and fundamental than the neighbour-modulated fitness approach [4–6]. Furthermore, Taylor & Frank [7] introduced a systematic methodology for constructing neighbour-modulated fitness models, which automatically accounts for all fitness effects. Taylor & Frank’s paper revolutionized how social evolution models are constructed, as it allowed modellers to make general (broadly applicable) models without the need for lots of model complexity [8,9]. There is no analogous systematic methodology for inclusive fitness, which has led to claims that inclusive-fitness models are more susceptible to mistakes because fitness effects are more easily missed [10]. For these reasons, neighbour-modulated fitness has emerged as the preferred modelling approach.

However, there are at least two problems associated with the neighbour-modulated fitness approach. First, by placing focus on the recipient of a behaviour, neighbour-modulated fitness misses the crux of most interesting problems in social evolution. Consider altruism as an example: it is an actor’s willingness to pay a cost to help another that is surprising, whereas a recipient’s willingness to accept help at the expense of another is not surprising at all. While we can use neighbour-modulated fitness as a calculating tool and rearrange our thinking to place focus on the actor [2,9], the necessary rearrangements are not always straightforward. The result is that the logic behind social behaviours carried out by actors remains hidden.

The second problem is that neighbour-modulated fitness is not something that can be optimized by individuals evolving by natural selection (a *maximand*) [11,12]. Therefore, even though the neighbour-modulated fitness approach can tell us when a given social behaviour will be favoured, it does not allow us to interpret the results of the model, in terms of actors striving to maximize some quantity. Explaining results in terms of individuals maximizing their fitness has a long tradition in behavioural and evolutionary ecology [13]. It is important because it allows us to understand adaptations, not just in terms of abstract selection pressures acting on populations, but in terms of decisions made by the individuals who wield the adaptations [14–16]. Another way of saying this is that explaining results in terms of individuals maximizing their fitness allows us to understand not only which traits are selected, but what they are selected *for* [17]?

The fundamental reason why neighbour-modulated fitness does not qualify as a maximand is that an individual’s neighbour-modulated fitness is affected by many independently evolving individuals [11]. For example, an individual can control who it helps but does not necessarily control who it receives help from. Generally speaking, then, an individual cannot maximize its own neighbour-modulated fitness, because part of its neighbour-modulated fitness lies outside of its control. A subsidiary reason why modern formulations of neighbour-modulated fitness do not qualify as maximands is that they make use of quantities that describe populations, which are not knowable or controllable by individuals. For instance, Taylor & Frank’s [7] neighbour-modulated fitness methodology makes use of allele invasion criteria. Other modern formulations of neighbour-modulated fitness similarly make use of population-level quantities, such as Rousset’s [18] use of allele fixation probabilities. A fitness measure can only qualify as a maximand if it is solely based on quantities that are under the control of individuals.

The standard ‘fix’ to this problem of neighbour-modulated fitness not being a maximand is to construct social evolution models using neighbour-modulated fitness, but then to interpret them *post hoc* using inclusive fitness [7,9,11,18]. Inclusive fitness is a possible maximand because it may lie fully under the control of an actor, meaning it is something that can be optimized by individuals evolving by natural selection [16,19,20]. However, no justification has been given for this fix, either in general, or in many of the specific instances where modellers have made use of it. An alternative and possibly more desirable approach would be to use a solely individual-centric inclusive fitness argument for modelling as well as interpretation, or at least to supplement a neighbour-modulated fitness argument with an inclusive fitness one that obtains the same optimized trait value, thereby justifying a maximization-based interpretation of results [21].

To bridge this gap between the analysis and interpretation of social evolution models, we present a systematic methodology for constructing social evolution models that are interpretable using maximands. To achieve this, our methodology articulates a fully individual-centric inclusive fitness argument, solving the two issues that prevent contemporary neighbour-modulated fitness formulations from being maximands (not fully individual-centric; influenced by multiple individuals). By articulating an inclusive fitness methodology that does not hinge on more basic neighbour-modulated fitness arguments, we demonstrate that inclusive fitness is no less fundamental than its neighbour-modulated fitness counterpart [21]. Our step-by-step approach tracks all relevant fitness effects, and is both mathematically and notationally simpler than modern neighbour-modulated fitness approaches, which should alleviate worries about inclusive fitness formulations being prone to errors, or difficult to implement [4,10]. In particular, our approach does not require differentiation, or any advanced mathematics, rendering it more accessible to biologists without formal training in mathematics or population genetics than neighbour-modulated fitness approaches.

On the one hand, our approach may be viewed as an alternative to the neighbour-modulated fitness methodology. One caveat to this perspective is that, unlike Taylor & Frank [7], we do not provide a formal proof that models generated with our approach will always lead to the same results as their population-genetic counterparts (i.e. single-locus ESS models). Our recommendation, then, is that whenever our methodology is used to obtain optimized trait values, these values should be checked against the optimized trait values obtained with an equivalent Taylor–Frank model. If the optimized trait values differ, it implies that a mistake has been made when implementing one of the two methodologies. On the other hand, our approach may be viewed, not as an alternative *per se*, but as a companion to neighbour-modulated fitness presentations. What we offer is a guide to extracting an actor-centric narrative that captures the adaptive significance of a behaviour or trait. This is the kind of valuable narrative that is not immediately apparent when applying neighbour-modulated fitness recipes. Taken together, our approach and that of Taylor & Frank [7] allow traits to be understood using ‘licenced anthopormorphism’, where the inclusive fitness argument tells us how to understand traits in terms of individuals maximizing their fitness for the trait (anthropomorphism), and the correspondence between optimized trait values obtained using each approach provides the justification (licence) for understanding traits in this way [22,23].

We structure the rest of the paper as follows. In §2, we outline the basic methodology for constructing inclusive fitness models when the population is homogeneous. We motivate our approach by drawing an analogy to sharing a pizza. In §3, we extend the methodology so that it can be used when the population is class-structured, i.e. subdivided into age classes, size classes etc. In §4, we extend the methodology further, so that it can be used when an action has consequences for multiple individuals spanning across different classes and at different points in time. In §5, we provide further discussion of our methodology, and its broader implications for social evolution theory. In the electronic supplementary material we illustrate our approach with numerous examples: help under synergy (electronic supplementary material, appendix B); help in a viscous population (electronic supplementary material, appendix C); Creel’s paradox (electronic supplementary material, appendix D); altruism between age classes (electronic supplementary material, appendix F); sex-ratio evolution (electronic supplementary material, appendix G).

## 2. Homogeneous populations

### (a) Sharing a pizza

Inclusive-fitness models are easier to understand if we first think about pizza. Suppose we divide a pizza among some number of competitors. We do so by giving larger slices to those whose competitive ability is above average and smaller slices to those competitive ability is below average. Specifically, if *w* is the average competitive ability and *w*_*i*_ is the competitive ability of individual *i*, then

$$(\text{fair share of pizza})\times \frac{w_{i}}{w},$$

expresses the size of the slice we pass to *i*. If *w*_*i*_ happens to be equal to the average *w*, then (of course) the slice we give to *i* is of average size. If, however, *w*_*i*_ differs from the average by some amount, say *δw*_*i*_, then the size of the slice given to *i* is

$$(\text{fair share of pizza})\times \left(1+\frac{\delta w_{i}}{w}\right).$$

**Figure 1.**
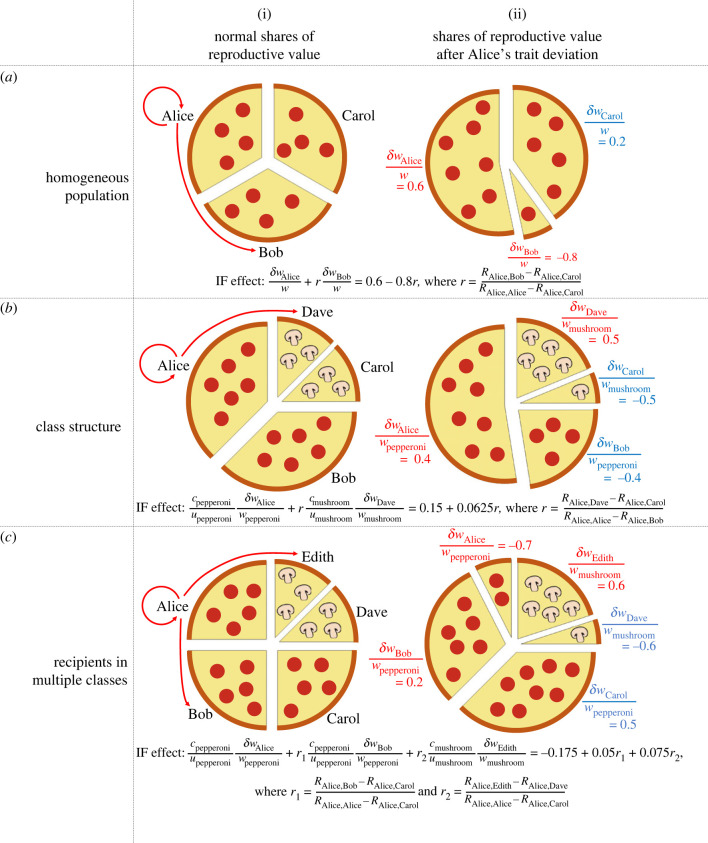
Calculating the inclusive fitness effect. A pizza represents the total reproductive value for a population. Alice (the focal individual) behaves in a way that changes the hunger (competitive ability) of herself and others (recipients), as shown by the red arrows in the (i) panels. Consequently, Alice and the other recipients get a share of pizza (ii) that differs from their normal (fair) share (i) by the proportion given in the red text in (ii) (primary changes in reproductive value). The remaining individuals get whatever pizza is left (ii), and this differs from their normal (fair) share (i) by the proportion given in the blue text in (ii) (compensatory changes in reproductive value). Alice’s behaviour is selected if the inclusive fitness effect is positive. (*a*) In homogeneous populations (all individuals like the same type of pizza), pizza is allocated on the basis of hunger (competitive ability). (*b,c*) In class-structured populations (individuals like different types of pizza), the pizza is split into segments with different toppings, and each individual in the population eats only one pizza topping. In general, there may be differences in the size of each segment (reproductive value ascribable to each class; the *c* terms) and the number of individuals who like each topping (number of individuals in each class; the *u* terms). Pizza is allocated on the basis of hunger (competitive ability) relative to the average hunger of the other individuals who eat that topping. We used: (*a*) *w*_Alice_ = 1.6, *w*_Bob_ = 0.2, *w*_Carol_ = 1.2; (*b*) *u*_pepperoni_ = 2, *u*_mushroom_ = 2, *w*_Alice_ = 1.4, *w*_Bob_ = 0.6, *w*_Carol_ = 0.5, *w*_Dave_ = 1.5; (*c*) *u*_pepperoni_ = 3, *u*_mushroom_ = 2, *w*_Alice_ = 0.3, *w*_Bob_ = 1.2, *w*_Carol_ = 1.5, *w*_Dave_ = 0.4, *w*_Edith_ = 1.6.

With this in mind, we understand *δw*_*i*_ / *w* as the factor by which *i*’s share of the pizza differs from the average. Importantly, if we give *i* a larger-than-average (resp. smaller-than-average) slice, then we must also give some number of its competitors a smaller-than-average (resp. larger-than-average) slice. Stated differently, *i*’s residual share of the pizza, represented by *δw*_*i*_ / *w*, must somehow be exactly compensated by the residual shares of its competitors. The kind of compensation that evidently occurs in contests over pizza is universal and (more to the point) is a central feature of our approach to inclusive-fitness modelling. With our approach, however, it is reproductive value, rather than pizza, that is shared among competitors (figure 1). The reproductive value of an individual refers to the expected fraction of the gene pool in the distant future that has descended from this individual; it captures an individual’s evolutionary success. Like a pizza, the total reproductive value of all individuals in the population is fixed: genes must have descended from ancestors carried by some individual, and no one individual can be ancestor to more than 100% of some future gene pool.

To make our approach work, we must assume that all deviations from the average competitive ability, *δw*_*i*_, are very small. This assumption allows us to proceed as if only one individual—the ‘focal actor’—expresses (or will ever express) the trait at a level that differs from the average. Thus, we can cast the focal actor as an innovator who ‘decides’ to express its trait in a way that deviates from the norm. Although the true consequences of the actor’s decision may be numerous and complicated, small deviations also mean that we can evaluate each consequence in isolation. So, if the focal actor (individual *i*) alters its fair share (henceforth, ‘normal’ share) of reproductive value as well as the share belonging to one of its neighbours (individual *j*), small deviations mean we can approximate the changes as *δw*_*i*_ / *w* and *δw*_*j*_ / *w*, respectively.

### (b) Method

We suppose an actor *i* changes its normal share of the total reproductive value of the population by a multiplicative factor *δw*_*i*_ / *w*, where *w* represents the normal competitive ability in the population. As an immediate result of the actor’s behaviour, we suppose some individual in the population, indexed by *j*, has its normal share of the total reproductive value altered. We use *δw*_*j*_ / *w* to represent the factor by which recipient *j* has its normal share of reproductive value changed.

Multiplicative factors *δw*_*i*_ / *w* and *δw*_*j*_ / *w* are weighted by measures of genetic similarity. For the moment, we measure genetic similarity using probabilities of identity by descent. Let *R*_*ij*_ be the probability that an allele drawn randomly from *i* is identical by descent to one drawn randomly from *j*, i.e. they descended from a common ancestor without mutation. Let *R*_*ii*_ be the probability that two alleles chosen randomly with replacement from *i* are identical by descent, so that *R*_*ii*_ = 1 for haploids and *R*_*ii*_ = (1 + *f*)/2 for diploids, where *f* is the inbreeding coefficient. With those measures of genetic similarity in hand, we define the primary inclusive-fitness effect of the actor’s behaviour as

2.1 $$R_{ii}\frac{\delta w_{i}}{w}+R_{ij}\frac{\delta w_{j}}{w}.$$

The term *δw*_*i*_ / *w* can be understood as a direct change in *i*’s success, realized through *i* itself and its offspring (i.e. *i*’s descendant kin) [1,24–26]. Often *δw*_*j*_ / *w* is understood as an indirect change in *i*’s success as it is realized through *i*’s non-descendant kin [1,24–26]. Sometimes, though, *j* may actually be the average member of some group of individuals that includes *i*, as would be the case, say, in a public-goods dilemma. In those instances *δw*_*j*_ / *w* combines direct and indirect effects.

The primary change in reproductive value due to the actor’s behaviour (line (2.1)) results in a compensatory change elsewhere in the population. Following Grafen [14], we suppose that individual *k* experiences the compensation that occurs, but, like individual *j*, *k* may be a randomly selected member of some group. We now define the secondary inclusive-fitness effect as

2.2 $$-R_{ik}\left(\frac{\delta w_{i}}{w}+\frac{\delta w_{j}}{w}\right).$$

This expression describes the ‘knock-on’ inclusive fitness effect of the actor’s deviant trait. The leading minus sign reminds us that the change in equation (2.2) is in a direction that is opposite the change expressed in equation (2.1). Thus, total change in reproductive value that arises immediately is exactly balanced.

The overall effect of an actor’s decision on its inclusive fitness is given by the sum of the primary and secondary effects given above. In other words,

2.3 $$R_{ii}\frac{\delta w_{i}}{w}+R_{ij}\frac{\delta w_{j}}{w}-R_{ik}\left(\frac{\delta w_{i}}{w}+\frac{\delta w_{j}}{w}\right),$$

gives the net effect of the actor’s decision to deviate from the norm. With an eye to simplifying line (2.3), we define

2.4 $$r=\frac{R_{ij}-R_{ik}}{R_{ii}-R_{ik}},$$

as the relatedness between the actor *i* and recipient *j* (see [27]), from the actor’s perspective. As an aside, our definition of *r* matches a geometric definition presented elsewhere [28], but we arrive at this definition by considering compensation (see electronic supplementary material, appendix A). It also makes it clear why some authors emphasize that competition between relatives must be considered whenever kin-selection is invoked, and not only when dispersal patterns keep related individuals together [25].

We now restate line (2.3) as

2.5 $$(R_{ii}-R_{ik})\underset{\mathrm{inclusive}-\mathrm{fitness}\;\mathrm{effect}}{\underbrace{\left(\frac{\delta w_{i}}{w}+r\frac{\delta w_{j}}{w}\right)}}.$$

Without loss of generality the leading term in the previous line is positive (or, at least, non-negative), and so the second term tells us whether the actor’s decision to deviate from the norm has increased or decreased its inclusive fitness. We call this second term the ‘inclusive-fitness effect’ owing to the deviant act carried out by the actor. If the inclusive-fitness effect is positive, then the actor’s deviation increases its inclusive fitness and is, therefore, favoured by selection. Conversely, if the inclusive-fitness effect is negative then the actor’s deviation decreases its inclusive fitness and is, therefore, disfavoured by selection. If the inclusive-fitness effect is zero, then the trait in question is at evolutionary equilibrium. The condition for the actor’s deviation to be favoured by selection—obtained by setting the inclusive-fitness effect to be greater than zero—is equivalent to ‘Hamilton’s Rule’ [1,29].

To summarize, formulating a model for the inclusive-fitness effect of changing a trait requires three things: (i) the identity of the actor (*i*), the recipient (*j*) and the individual who will compensate for changes in reproductive value (*k*); (ii) the primary effects of the actor’s behaviour, expressed as proportional changes to an individual’s share of total reproductive value (*δw*_*i*_ / *w* and *δw*_*j*_ / *w*); (iii) the coefficient of consanguinity normally found between *i* and itself (*R*_*ii*_), between *i* and *j* (*R*_*ij*_) and between *i* and *k* (*R*_*ik*_). We use the third item in this list to formulate relatedness as *r*, which then weights primary effect *δw*_*j*_ / *w* in the standard way. Our use of the term ‘normally’ in the third item signals that it is enough to use estimates that assume no deviant actions have taken place.

A word of caution for those looking to use equation (2.5) to classify social behaviours is appropriate here. Social behaviours are often classified according to direct and indirect fitness effects [1,26]. Mutually beneficial behaviours are those that are beneficial to the actor (positive direct fitness effect) and beneficial to non-actor recipients (positive indirect fitness effect). Spiteful behaviours are costly to both the actor and non-actor recipients (negative direct and indirect fitness effects). Altruistic behaviours are costly to the actor but beneficial to non-actor recipients. Selfish behaviours are beneficial to the actor but costly to non-actor recipients. Equation (2.5) may not separate indirect effects from direct ones in a way that allows the trait in question to be classified accurately. Readers interested in determining whether a given trait is altruistic, spiteful etc. may have to look in greater detail at the net effects on individuals captured in both primary and secondary changes [30].

In the electronic supplementary material, we illustrate how to use the methodology for homogeneous populations with three examples: help under synergy (electronic supplementary material, appendix B); help in a viscous population (electronic supplementary material, appendix C); and Creel’s paradox (electronic supplementary material, appendix D). The reader who is interested in more than just an overview of our approach should review those examples before proceeding.

## 3. Class structure

Two individuals can have the same genotype at a particular locus, yet be qualitatively different. In these cases, we say that the individuals belong to different classes. Those classes could be female/male, large/medium/small, young/old, etc. To model these class-structured populations, we must modify the model-building steps presented earlier.

We suppose the actor *i* is in class *X* and its actions affect recipient *j* in class *Y*. We use *c*_*X*_ and *c*_*Y*_ to represent the fractions of total reproductive value earmarked for individuals in class *X* and class *Y*, respectively. Returning to the pizza analogy, *c*_*X*_ might be the fraction of the pizza reserved for those who like pepperoni, and *c*_*Y*_ might be the fraction reserved for those who like mushrooms (figure 1*b*,*c*). Given *u*_*X*_ and *u*_*Y*_ to represent the number of individuals in class *X* and class *Y*, respectively, we recognize *c*_*X*_/*u*_*X*_ and *c*_*Y*_/*u*_*Y*_ as a normal share of reproductive value within class *X* and class *Y*, respectively. Alternatively, *c*_*X*_/*u*_*X*_ is the share of the pizza given to the average pepperoni-lover, and *c*_*Y*_/*u*_*Y*_ is the share given to the average mushroom-lover.

For the class-structured population, we describe the primary effect as

3.1 $$R_{ii}\frac{c_{X}}{u_{X}}\;\frac{\delta w_{X,i}}{w_{X}}+R_{ij}\frac{c_{Y}}{u_{Y}}\;\frac{\delta w_{Y,j}}{w_{Y}}.$$

Compensation in a class-structured population happens within each class, analogously to how altering the size of the pizza slice given to a pepperoni-lover affects only other pepperoni-lovers (this holds even when there is competition among classes; see the example in electronic supplementary material, appendix E). Thus, the secondary effect is

3.2 $$-R_{ik}\;\frac{c_{X}}{u_{X}}\;\frac{\delta w_{i,X}}{w_{X}}-R_{im}\;\frac{c_{Y}}{u_{Y}}\;\frac{\delta w_{Y,j}}{w_{Y}},$$

where individual *k* is now the average member of class *X* who compensates for the actor’s change in competitive ability, and individual *m* is the average member of class *Y* who compensates for the recipient’s change in competitive ability. We calculate relatedness in a class-structured population as

3.3 $$r=\frac{R_{ij}-R_{im}}{R_{ii}-R_{ik}},$$

and so we arrive at

3.4 $$(R_{ii}-R_{ik})\underset{\text{inclusive - fitness effect}}{\underbrace{\left(\frac{c_{X}}{u_{X}}\;\frac{\delta w_{X,i}}{w_{X}}+r\frac{c_{Y}}{u_{Y}}\;\frac{\delta w_{Y,j}}{w_{Y}}\right)}}.$$

The sign of the second term in the previous line tells us when the actor’s deviation increases its inclusive fitness and when its deviation decreases its inclusive fitness.

To formulate a model for the inclusive-fitness effect of changing a trait in a class-structured population we need to know the items previously listed, alongside another three things: (i) the fraction of total reproductive value normally allocated to each class (*c*_*X*_ and *c*_*Y*_); (ii) the number (or relative number) of individuals normally found in each class (*u*_*X*_ and *u*_*Y*_); and (iii) the coefficient of consanguinity normally observed between *i* and individual *k* from class *X* (*R*_*ik*_), and between *i* and individual *m* from class *Y* (*R*_*im*_). Again, we use the term ‘normally’ to signal that we use estimates that assume no deviant behaviour has taken place.

We typically need a model for the normal demographics of a class-structured population before formulating a kin-selection model for evolution therein. The asymptotic behaviour of the demographic model gives us the information we need to make sensible statements about *c*_*X*_ and *u*_*X*_, as seen in the examples in the electronic supplementary material, appendix F & G. The total reproductive value of a class *c*_*X*_ is calculated as the probability that the ancestor of a random gene in the distant future resides in a class *X* individual today [31,32]. In some cases, the *c*_*X*_/*u*_*X*_ weights are set equal to one another and authors proceed with appropriate caution. For example, they might emphasize that fitness payoffs received by individuals are uncorrelated with the class to which they belong [33]. In rare instances, the expression *c*_*X*_/*u*_*x*_ can be viewed as a parameter in a so-called ‘open’ model [34].

In the electronic supplementary material, we illustrate how to use the methodology for class-structured populations with an example of altruism between age classes (electronic supplementary material, appendix F). As before, we encourage the reader who is interested in more than just an overview of our approach to review this example before proceeding.

## 4. Further extensions

Our approach is easily generalized to include the possibility that recipients occur in different classes, say *Y*_1_ and *Y*_2_. Combining primary and secondary effects leads us to

4.1 $$\begin{array}{rl} & (R_{ii}-R_{ik})\frac{c_{X}}{u_{X}}\;\frac{\delta w_{X,i}}{w_{X}}+(R_{ij_{1}}-R_{im_{1}})\frac{c_{Y_{1}}}{u_{Y_{1}}}\;\frac{\delta w_{Y_{1},j_{1}}}{w_{Y_{1}}}\\ & \;\;\;+(R_{ij_{2}}-R_{im_{2}})\frac{c_{Y_{2}}}{u_{Y_{2}}}\;\frac{\delta w_{Y_{2},j_{2}}}{w_{Y_{2}}}, \end{array}$$

where individual *m*_1_ compensates for class-*Y*_1_ recipient *j*_1_, and individual *m*_2_ compensates for class-*Y*_2_ recipient, *j*_2_. Using $r_{1}=(R_{ij_{1}}-R_{im_{1}})/(R_{ii}-R_{ik})$ along with *r*_2_ = $\left(R_{ij_{2}}-R_{im_{2}})/(R_{ii}-R_{ik}\right)$, we obtain

4.2 $$\frac{c_{X}}{u_{X}}\;\frac{\delta w_{X,i}}{w_{X}}+r_{1}\;\frac{c_{Y_{1}}}{u_{Y_{1}}}\;\frac{\delta w_{Y_{1},j_{1}}}{w_{Y_{1}}}+r_{2}\;\frac{c_{Y_{2}}}{u_{Y_{2}}}\;\frac{\delta w_{Y_{2},j_{2}}}{w_{Y_{2}}}\;$$

as the inclusive-fitness effect. Further generalization to more than two classes is obvious.

One might wonder how the inclusive-fitness effect is formulated when actors belong to different classes. We neglect generalization in this direction because we assume that an actor knows the class to which it belongs and expresses the deviant trait only when it belongs to class *i*. In other words, we assume that the allele that underlies deviant actor behaviour is expressed conditional on membership in class *i*.

In some cases, two distinct individuals in the same class *Y*, say *j* and ℓ, are affected by the actor’s deviant behaviour. Generalization of the method to this case is also obvious, but the application is more complicated when *j* and ℓ are affected by the actor’s decision at different points in time. Suppose the actor’s decision affects individual *j* first and individual ℓ, later. To emphasize the order, we decorate quantities associated with ℓ with prime; for instance individual *m* compensates for *j* and individual *m*′ compensates for ℓ. Combining primary and secondary effects gives us

4.3 $$\frac{c_{X}}{u_{X}}\;\frac{\delta w_{X,i}}{w_{X}}+r\;\frac{c_{Y}}{u_{Y}}\;\frac{\delta w_{Y,j}}{w_{Y}}+r^{\prime }\;\frac{c_{Y}}{u_{Y}}\;\frac{\delta w_{Y,\ell }^{\prime }}{w_{Y}^{\prime }}$$

as the inclusive-fitness effect, where *r* = (*R*_*ij*_ − *R*_*im*_)/(*R*_*ii*_ − *R*_*ik*_) and *r*′ = (*R*_*i*ℓ_ − *R*_*im*′_)/(*R*_*ii*_ − *R*_*ik*_). Further generalization to more than two recipients in the same class, or to multiple recipients in each of several classes, is obvious.

We extend our pizza analogy to capture line (4.3) by imagining that an individual’s competitive ability is actually the product of, say, how aggressive it is (*w*_*j*_) and how hungry it is (*w*_*j*_′). A meek and hungry individual, for example, may be as competitive as a belligerent sated one. Because our method assumes that deviations are small, for the purposes of evaluating the actor’s influence on *j*’s aggression (*δw*_*j*_), we assume that aggression alone determines how the pizza is shared; for the purposes of evaluating the actor’s influence on *j*’s hunger (*δw*_*j*_′), we assume that hunger alone determines how the pizza is shared. While the analogy, here, imagines that the same individual *j* = ℓ is affected in two distinct ways, in general *j* need not be the same as ℓ, which leads to the formulation in line (4.3).

We illustrate the application of equation (4.3) in appendix G of the electronic supplementary material. In that appendix, we focus on the evolution of the sex ratio. While sex-ratio evolution in group-structured populations has provided some of the strongest support for Hamilton’s theory, it is often difficult to model without a systematic methodology (see p. 143 of [9]; [35]). Indeed, before systematic approaches to kin-selection modelling were available, discussions about the mechanisms of sex-ratio evolution were routinely marked by controversy [36,37]. Applying our method to the study of sex-ratio evolution, therefore, is an important litmus test: a set of steps that purport to be a method of kin-selection modelling must cope with the challenge of sex-ratio evolution. As we show in electronic supplementary material, appendix G, our approach builds an argument that is less cumbersome than the one used in neighbour-modulated fitness models.

Our approach to deriving the inclusive-fitness effect for sex-ratio problems communicates a prediction in a way that is less technical than earlier inclusive-fitness formulations. In particular, we are not concerned with the population-genetic details that appear elsewhere [32,38,39] when developing a prediction. Of course, we are indebted to previous authors, as their painstaking work has allowed us to focus on a more intuitive approach to modelling.

## 5. Discussion

We have articulated a systematic methodology for constructing inclusive-fitness models (for a summary, see box 1).

Box 1. **Steps in making an inclusive-fitness model.**

Those interested in building an inclusive-fitness model will benefit by working through the three steps below before attempting to use equations (2.5), (3.4), (4.2) and (4.3). The steps below will also be helpful to those who have already constructed a model using the neighbour-modulated fitness approach [7] but are looking to uncover an inclusive-fitness narrative that matches their model assumptions.

1.  **Establish the life-history details and demography of the model organism.** At this stage, it is helpful to ask several questions: when does birth happen? When does death occur? Who competes with whom? Who mates with whom? Is the population structured in any way? Do individuals migrate from one location to another? If so, then when does this migration occur? You need only focus on what happens ‘normally,’ that is, when all individuals of the same class (same age, sex, size etc.) behave in exactly the same manner. Where applicable, you should have enough information at this stage to calculate quantities like *c*_*X*_ and *u*_*X*_, as well as relatedness (see electronic supplementary material, appendices).
2.  **Identify the point in time when the primary social interaction occurs, and establish the immediate fitness consequences for actor and recipient**. The primary social interaction represents the first deviation from what ‘normally’ occurs in the population. For instance, an actor, in a population that is normally selfish, might decrease its ability to compete for reproductive value by *C* × 100% to increase that of a recipient by *B* × 100%. Alternatively, an actor who would normally produce equal numbers of daughters and sons might, instead, convert a son into a daughter, effectively erasing the existence of one recipient and spawning another. Establishing exactly when the primary fitness changes felt by actor and recipient, respectively, take place is important as the timing determines secondary changes (Step 3).
3.  **Identify all points in time, following the primary interaction, at which those involved in the primary interaction compete for reproductive value.** Compensation takes place at these points in time. If a primary player’s ability to compete for reproductive value was reduced (e.g. because it made an altruistic donation to another), then its competitor(s) at this time will benefit. Conversely, if a primary player’s ability to compete for reproductive value was improved (e.g. because it received an altruistic donation), then its competitor(s) at this time will be disadvantaged. You should establish the ‘normal’ competitive ability of all competitors at the (potentially various) points in time you identify. At this stage, it is also helpful to ask questions about the scale at which competition occurs: are individuals competing with individuals chosen at random from the population (global scale)? Alternatively, is competition occurring only among individuals found in the same neighbourhood (local scale)? The scale at which this competition occurs determines the relatedness between the actor and the individuals who make up for, or are displaced by, primary players in the scramble for reproductive value.

Our methodology examines whether a given deviant behaviour will be favoured. We assume that the deviant behaviour is only slightly different from the behaviour exhibited by the incumbent population (*δ*-weak selection), which means that the deviant behaviour will be favoured if it increases the inclusive fitness of the actor (is associated with a positive inclusive-fitness effect) [40]. We assume that the total reproductive value of the population is fixed [16,41]. The inclusive-fitness effect is then obtained by summing the primary (immediate) and secondary (compensatory) changes in reproductive value experienced by each individual as a consequence of the focal actor’s deviant behaviour, where each of these changes in reproductive value is weighted by the coefficient of consanguinity between the actor and the affected individual. Additionally, we show how the inclusive-fitness effect can be obtained more simply by subsuming several coefficients of consanguinity into a single relatedness parameter that incorporates density dependence [27,28,39,42–44]. Optimized trait values are obtained as the trait values from which slight behavioural deviations are disfavoured [45].

Our assumption of small behavioural deviations (*δ*-weak selection) is commonly taken in social evolution models [7]. It is justified empirically by the observation that the vast majority of traits are controlled by many mutations of small effect [46]. Fisher’s [31] geometric model explains why traits tend to evolve to be governed by mutations of small effect. Fisher examined a population that is displaced from an optimum in multi-dimensional phenotype space, and allowed to return to the optimum through random gene substitutions. Fisher [31] found that very small mutations have a 50% chance of bringing the population towards the optimum, but the larger the size of the mutation, the greater the chance of overshooting in some dimension, and the lower the resulting chance of bringing the population towards the optimum. Overshooting is particularly likely when the population lies close to the optimum, as is the case for adaptive (optimized) behaviours [31,45]. The upshot is that most mutations that fix in a population differ only slightly from the mutations that they displaced, especially for adaptive behaviours, which are the main focus of behavioural and evolutionary ecology [31,45,47]. Consequently, behavioural deviations tend to be small for traits of interest.

One important consequence of our assumption of small behavioural deviations (*δ*-weak selection) is that it generates *additivity* in fitness effects. In other words, under *δ*-weak selection, whenever two deviant individuals interact, multiplicative effects on reproductive value are approximated by zero [40]. Consequently, the effect that a deviant individual (actor) has on the reproductive value of other individuals (recipients) is uninfluenced by the genotypes of the recipients. This means that the inclusive-fitness effect associated with a deviant behaviour is solely a property of the individual exhibiting the behaviour (actor control). With actor control, the condition for a given behaviour to be favoured is the same as the condition for an actor to increase its inclusive fitness for the behaviour, which means the evolved behaviour can be interpreted as an adaptive ‘choice' made by individuals to improve their inclusive fitness for the behaviour [11,16,19]. Therefore, the assumption of small behavioural deviations is essential for applying the maximand-based approach of behavioural and evolutionary ecology to social evolution problems.

It is worth considering where we would be left if we did not assume small behavioural deviations (*δ*-weak selection). First, multiplicative effects on reproductive value would not in general be approximated by zero (non-additivity), meaning the ‘inclusive-fitness effect’ associated with a deviant behaviour would not solely be a property of the individual exhibiting the behaviour, as it would depend on the genotypes of the recipients of the behaviour (lack of full actor control). Without actor control, the condition for a given behaviour to be favoured would involve requirements about the genotypes of recipients, meaning satisfaction of the condition would not necessarily imply that the actor is improving its own inclusive fitness for the behaviour. This precludes a familiar maximand-based interpretation of the results. These points are true of inclusive fitness in a broad sense—not just the formulation of it in our methodology [16,19,20,48].

The inability of inclusive fitness to handle large behavioural deviations, with their non-additively combining fitness effects, is usually reported as a limitation of inclusive-fitness theory, with the implication being that inclusive-fitness theory would be a better and more encompassing theory if it could handle such cases [47,48]. However, it may be a red herring to even want to apply inclusive fitness in many of these cases, because the condition for the deviant behaviour to be favoured will depend on what multiple individuals (agents) are doing, not just one (frequency dependence). Therefore, we should not expect non-additively combining traits to evolve according to a function that is solely ascribable to one individual, such as inclusive fitness. Instead, the evolved trait value will reflect the coevolution of multiple agents at cross-purposes, where each individual may strive to maximize some personal function, but where the pulls and pushes of the coevolutionary process are unlikely to lead to the outright maximization of any one personal function.

Therefore, we shouldn’t generally expect to find single-actor maximization principles in non-additive scenarios (for an alternative perspective see [49]). Non-additive scenarios involve multiple actors, so we suggest that they be analysed with approaches that simultaneously consider multiple agents, like game theory. However, we re-iterate that the empirical observation that the vast majority of traits are controlled by many mutations of small effect, explained by Fisher’s geometric model, gives us reason to think that genuinely non-additive scenarios will be rare. Even apparently large behavioural differences (such as those often found between reproductives and helpers in animals) may be governed by small-effect genes dictating a probability of exhibiting one behaviour or another [20,31,45,48].

We have articulated a set of steps for constructing inclusive-fitness models, but we are certainly not the first to develop an approach to inclusive-fitness modelling. Previous work has clearly demonstrated that Hamilton’s [1] ideas extend to sexual populations, class-structured populations and populations experiencing random demographic fluctuations [32,50–52]. This previous work is based on neighbour-modulated fitness versions of the Price equation [53] or other population-genetic formulations, and so is naturally recipient-centred. It is not surprising, then, that the predominant step-by-step approach to building kin-selection models, outlined by Taylor & Frank [7], has adopted a similar recipient-centred perspective. While there is nothing incorrect about formulating a model in a recipient-centred way before re-organizing calculations to create an actor-centred narrative, doing so in an algorithmic manner is more complicated. Moreover, the focus placed on the recipient-centred approach tacitly suggests it is the more fundamental perspective. The emphasis we place on the actor, here, shows that inclusive-fitness can stand alone. In light of Taylor *et al.*’s [2] finding that recipient-centred accounting and actor-centred accounting often lead to the same predictions, it is not surprising that a step-by-step approach for the latter exists. That said, the ideas found in Taylor *et al.* [2], and elsewhere [9,14,27,42,54], fall short of a practicable, general-purpose method.

The methodologies presented here and by Taylor & Frank [7] predict optimized trait values, and to achieve this while keeping things as simple as possible, they sacrifice an account of how genotype frequencies change over generations. An implication of this is that, in general, the methodologies provided here and by Taylor & Frank [7] cannot: (i) identify stable genetic polymorphisms; (ii) track the evolution of exact statistical associations between alleles; or (iii) examine evolutionary stability, which is necessary in order to distinguish between evolutionarily stable strategies and branching points [55]. To obtain these kinds of insights, we would need to make more assumptions about genetic architecture, which would mean that the conclusions are likely to hold less generally. Systematic methodologies for constructing such detailed models in social evolution have been developed [56].

Foundational work in population genetics has found that the ‘inclusive-fitness effect’ of a given strategy successfully predicts allele frequency change [18,32,38,39,56]. This has two implications for our approach to inclusive-fitness modelling. First, it supports our decision to focus entirely on individual-level quantities like the ‘inclusive fitness effect’ of a given strategy, while keeping the underlying genetics implicit. In other words, it means we can be confident that our approach will not generate predictions that disagree with approaches based on population genetics. Second, by demonstrating that the ‘inclusive fitness effect’ of a given strategy is improved by natural selection, it strongly implies (implicitly) that an individual’s inclusive fitness for the strategy will be continually increased and ultimately maximized. This complements work explicitly linking gene frequency change to individual inclusive fitness maximization, in model settings that are general in different ways, sacrificing either dynamic sufficiency [19] or some behavioural flexibility on behalf of the actors [16,20].

Our approach relies on the fact that the total reproductive value of the population is fixed. While this fact has been recognized numerous times, it has not been used to drive a more general approach to inclusive-fitness modelling [27,42,54,57,58]. The attention we give to the fixed nature of total reproductive value is, equivalently, attention given to reproductive value as a relative measure of success. In this way, we are also motivated by foundational work showing that relative reproductive value is maximized by natural selection in a wide class of models [16,41,59–62].

Finally, our methodology will predict the same optimized trait values as Taylor & Frank [7] under a standard set of mathematical assumptions, and shares many of the advantages of Taylor & Frank’s [7] approach. For instance, the methodologies presented here and by Taylor & Frank [7] provide systematic ways to count up fitness effects on one individual (either an actor or recipient), eliminating the risk of ‘double counting’ fitness effects. Double counting is a common cause of error in informal reasoning about social evolution [63]. Additionally, our methodology has the advantages over Taylor & Frank [7] of: (i) being a fully actor-centric argument, justifying interpretation in terms of individual fitness maximization; (ii) not requiring a full fitness function, which means it can sometimes work with fewer assumptions, and streamline the mathematical argument, as shown by our sex ratio example in electronic supplementary material, appendix G; (iii) requiring no differentiation or complex mathematics. Admittedly, some readers may not be moved enough by the advantages we list to abandon recipient-centred approaches altogether. For those readers, we wish to emphasize that our approach will still prove useful for uncovering the inclusive-fitness narrative hidden in the neighbour-modulated fitness equations.

## Data Availability

The data are provided in electronic supplementary material [64].
